# An investigation of relationships between body compassion, social physique anxiety and physical appearance perfectionism in young people from Iran

**DOI:** 10.1186/s40337-023-00807-x

**Published:** 2023-06-05

**Authors:** Abbas Abdollahi, K. D. V. Prasad, Nasser Said Gomaa Abdelrasheed, Shadia Hamoud Alshahrani, Sarah Jawad Shoja, Ghaidaa Raheem Lateef Al-Awsi, Edwin Gustavo Estrada-Araoz, Nermeen Singer, Andrés Alexis Ramírez-Coronel, Yasser Fakri Mustafa, A. Heri Iswanto

**Affiliations:** 1 Department of Counseling, Faculty of Education and Psychology, Alzahra University, Tehran, Iran; 2 Symbiosis Institute of Business Management, Hyderabad, India; 3 Symbiosis International (Deemed University), Pune, India; 4 Department of Education, Dhofar University, Salalah, Sultanate of Oman; 5 Medical Surgical Nursing Department, King Khalid University, Khamis Mushate, Saudi Arabia; 6 College of Health and Medical Technology, Al-Ayen University, Nasiriyah, Iraq; 7 Department of Radiological Techniques, Al-Mustaqbal University College, Hillah, Babylon, 51001 Iraq; 8 Academic Department of Education and Humanities, Universidad Nacional Amazónica de Madre de Dios, Puerto Maldonado, Peru; 9 Media and Children’s Culture, Ain Shams University, Cairo, Egypt; 10 Laboratory of Psychometrics, Comparative Psychology and Ethology (LABPPCE), Universidad Católica de Cuenca, Cuenca, Ecuador; 11 Universidad CES, Medellín, Colombia; 12 Department of Pharmaceutical Chemistry, College of Pharmacy, University of Mosul, Mosul, 41001 Iraq; 13 Public Health Department, University of Pembangunan Nasional Veteran Jakarta, Jakarta, Indonesia

**Keywords:** Physical appearance perfectionism, Social physique anxiety, Body compassion, Undergraduates, Moderator

## Abstract

**Background:**

Previous studies have shown that physical appearance perfectionism could play an important role in social physique anxiety; however, the moderating role of body compassion has not been studied. The current study aims to explore the moderating role of body compassion in the relationship between physical appearance perfectionism and social physique anxiety in undergraduate students.

**Methods:**

A sample of 418 undergraduates (n = 418; 217 female and 201 males) from three universities in Tehran, Iran completed online questionnaires measuring physical appearance perfectionism, body compassion and social physique anxiety.

**Results:**

The results of structural equation modeling showed that physical appearance perfectionism (β = 0.68, *p* < 0.001) positively predicted the social physique anxiety and body compassion negatively predicted (β = − .56, *p* < 0.001) the social physique anxiety in undergraduate students. A multi-group analysis showed that body compassion acted as a moderator between physical appearance perfectionism and social physique anxiety.

**Conclusions:**

The results suggested that individuals with greater levels of physical appearance perfectionism are more likely to experience social physique anxiety. Also, the results suggested that individuals who were at a high level of the body-compassion group experienced lower levels of social physical anxiety if they also had high levels of physical appearance perfectionism. Therefore, body-compassion acted as a protective role in the relationship between physical appearance perfectionism and social physique anxiety.

## Abstract (plain language summary)

Anxiety over one's physical appearance in social situations is known as social physique anxiety. One of the variables that plays an important role social physique anxiety is physical appearance perfectionism. General perfectionism, body dissatisfaction, the symptoms of eating disorders and muscle dysmorphia and obsessive exercise are all linked to physical appearance perfectionism. The results of the current study showed individuals with high levels of physical appearance perfectionism are more likely to experience social physique anxiety. In the current study, we tested the moderating role of self-compassion in the relationship between physical appearance perfectionism and social physique anxiety. Body compassion is characterized by diffusion, common humanity, and acceptance of one's own body as opposed to being judgmental, critical, isolated, and over-identifying with unpleasant experiences and emotions. The findings showed in the group with high body- compassion, the relationship between physical appearance perfectionism and social physique anxiety was not significant, which means that body-compassion played a buffering role in this relationship.

## Introduction

We live in a culture where having a perfect physical appearance is highly valued. Peers, friends, family members, and the media place high beauty standards on individuals, inculcating looks as a symbol of success, happiness, and being loved and admired by others [1]. The more people internalize culturally prescribed physical ideals, and the farther away from these physical ideals they are, the more likely these individuals are to experience social physique anxiety. Social physique anxiety is a psychological variable that represents a person’s perceived concern about how she or he appears in situations where she or he believes others are evaluating their figures and size of their body [2, 3]. Studies have shown that females with high levels of social physique anxiety reported higher levels of drive for thinness, whereas males with high levels of social physique anxiety reported higher levels of drive for muscularity [3, 4]. Social physique anxiety is associated with several variables that play an important role in individuals’ mental health, such as self-esteem [5], body satisfaction [6], eating disorders [7], and physical activity [4]. As such, it is important to study factors that play an important role to the contribution and development of social physique anxiety.

A specific variable that plays an important role in social physique anxiety is physical appearance perfectionism [8, 9]. Previous studies have shown that individuals with high physical appearance perfectionism believe that their appearance is flawed and compare their appearance with others. These individuals are more likely to experience higher levels of social physique anxiety if their appearances are far from the physical ideals of society. Individuals with physical appearance perfectionism have cognitive distortions of their appearances and have negative evaluations of their appearances compared to others, and feel more vulnerable when they overestimate the attractiveness of others [10]. Stoeber and Stoeber [11] investigated the prevalence of perfectionism in 22 different areas of undergraduate students' lives and found that physical appearance perfectionism was the fourth most prevalent area in which students reported. 40% of students showed to be physical appearance perfectionists, suggesting physical appearance perfectionism is one of the more prevalent areas of perfectionism in undergraduate students. Physical appearance perfectionism was found to be negatively connected to body satisfaction and physical self-esteem. This showed that physical appearance perfectionism is positively related to social physique anxiety and appearance stress in cosmetic surgery patients [12, 13].

The linear association between physical appearance perfectionism and social physique anxiety has been explored in the past studies [6, 8]; however, the moderating role of body compassion in the relationship between these two variables has yet to be investigated. Body compassion is characterized by diffusion, common humanity, and acceptance of one's own body as opposed to being judgmental, critical, isolated, and over-identifying with unpleasant experiences and emotions [14]. The three components of self-compassion [15] are combined in body compassion, with an emphasis on body image. The Body Compassion Scale (BCS) developed by Altman et al. [14] was used in the current study to assess how people perceive their body's flaws and limitations. The BCS measures three subscales namely diffusion, common humanity, and acceptance. Diffusion refers to the ability of individuals to perceive their body imperfections and limitations and to diffuse these defects from their own self. Common humanity refers to the individuals' ability to understand the imperfect and deficient aspects of their bodies as part of the broader human experience. Acceptance refers to the acceptance of body-related unpleasant thoughts, perceptions, and feelings in a generous and kind manner, instead of adopting a self-judgmental attitude [14]. It is possible that individuals with higher levels of body compassion have a healthier body image, less body dissatisfaction, and less body embarrassment. Such individuals are more likely to experience greater body appreciation and body flexibility [16, 17]. People with high levels of body compassion respond to their body limitations and deficits with kindness, acceptance, and non-judgmental attitudes [16, 18, 19]. These attitudes help individuals experience less negative feelings and social physique anxiety. Based on this information, the first goal of this study was to examine the relationships between physical appearance perfectionism, body compassion, and social physique anxiety among undergraduate students in Tehran, Iran. The second goal of this study was to explore whether body compassion plays a moderating role in the relationship between physical appearance perfectionism and social physique anxiety.

## Methods

### Participants

The participants were (n = 418; 217 female and 201 males) recruited from undergraduates and employed from three universities in Tehran, Iran. The approximate number of eligible participants in Tehran University, Allameh Tabatabai University and Azad University were 400, 500 and 600, respectively. The number of participants who completed the questionnaires from Tehran University, Allameh Tabatabai University and Azad University were 110, 123 and 185, respectively. In terms of fields of study, the number of participants in psychology and counselling were 280 and 138, respectively. The participant's age ranged from 18 to 25 (M = 23.74 and SD = 2.14). Female mean age and male mean age were 22.81 (SD = 1.81) and 23.91 (SD = 2.11). In terms of educational levels, 92 (21.8%) of the participants were in the first year of university degree, 193 (46.3%) were in the second year of university degree, 99 (23.8%) were in the third year of university degree, and 34 (8.1%) were in the last year of university degree. In terms of marital status, 321 (76.8%) of participants were single and 97 (23.2%) of participants were married. The means of body mass index for females and males were 20.14 (SD = 2.17) and 23.15 (SD = 2.41). The means and standard deviation values of studied variables and items are presented in Table 1.

**Table 1:** Means and standard deviations of items and variables

		Mean	Standard deviation
*Social physique anxiety*		29	2.8
SPAA1	I wish I wasn’t so up-tight about my physique or figure	3.4	1.1
SPAA2	There are times when I am bothered by thoughts that other people are evaluating my weight or muscular development negatively	3.5	1.2
SPAA3	Unattractive features of my physique or figure make me nervous in certain social settings	4	0.98
SPAA4	In the presence of others‚ I feel apprehensive about my physique or figure	3	1.3
SPAA5	I am comfortable with how fit my body appears to others	3.5	0.88
SPAA6	It would make me uncomfortable to know others were evaluating my physique or figure	3.8	0.66
SPAA7	When it comes to displaying my physique or figure to others‚ I am a shy person	4	0.55
SPAA8	I usually feel relaxed when it’s obvious that others are looking at my physique or figure	4.1	0.65
SPAA9	When in a bathing suit‚ I often feel nervous about how well-proportioned my body is	3.9	0.54
*Body compassion scale*			
Diffusion		31.23	3.23
DF1	When I feel frustrated with my body’s inability to do something, I tend to feel separate and cut off from other people	3.2	0.77
DF2	When I think about my body’s inadequacies, it tends to make me feel more separate and cut off from other people	3.4	0.73
DF3	When I fail at some form of physical activity that is important to me, I tend to feel alone in my failure	4	0.83
DF4	When my body fails at something important to me, I become consumed by feelings of inadequacy	2.9	0.97
DF5	When my body is not responding the way I want it to, I tend to be tough on myself	3.6	1.3
DF6	When I wish some aspect of my body looked different, it feels like no one else understands my struggle	4.1	0.87
DF7	When I have physical symptoms, illness, or injury, it tends to make me feel more separate and cut off from other people	3	0.1.2
DF8	When I notice aspects of my body that I do not like, I get down on myself	3.7	0.98
DF9	When I am feeling physically uncomfortable, I tend to obsess and fixate on everything that is wrong	3.7	1.2
Common humanity		29.87	3.87
CH1	When I am frustrated with some aspect of my appearance, I try to remind myself most people feel this way at some time	3	1.8
CH2	When I doubt my ability to do a new physical activity, I try to remind myself that most people also feel this way at some point	4	0.74
CH3	When I feel out of shape, I try to remind myself that most people feel this way at some point	3.8	0.91
CH4	I try to see my body’s failings as something everyone experiences in one way or another	4	0.74
CH5	When I am injured, ill, or have physical symptoms, I remind myself that there are lots of other people in the world feeling like me	4.1	0.55
CH6	When I feel frustrated with my body’s inability to do something, I try to remind myself that most people in my condition feel this way at some point	4.2	0.69
CH7	When I feel my body is inadequate in some way, I try to remind myself that feelings of inadequacy are shared by most people	4	0.54
CH8	When I am at my lowest during times of physical symptoms, illness, or injury, I know I am not alone in feeling this way	4	1
CH9	When I am concerned if people would consider me good-looking, I remind myself that most everyone has the same concern	4	1
Acceptance		19.12	3.9
AC1	I am accepting of my looks just the way they are	3.1	1.6
AC2	I am accepting of the way I look without my clothes on	3.3	0.67
AC3	I feel okay in my body	3	0.79
AC4	I am tolerant of my body’s flaws and inadequacies	3.6	1.5
AC5	I am tolerant of the way my clothes fit me	3	1.92
*Physical appearance perfectionism scale (worry about imperfection)*		21.23	2.9
WI1	I am not satisfied with my appearance	2.5	2.1
WI2	I am never happy with my appearance no matter how I dress	2.9	2.4
WI3	I worry that my appearance is not good enough	3.1	1.8
WI4	I wish I could completely change my appearance	2.5	1.9
WI5	My appearance is far from my expectations	3.4	1.1
WI6	I worry about others’ being critical of my appearance	3.5	1.4
WI7	I often think about shortcomings of my appearance	3.6	1.9

### Procedure

The current study aims were reviewed and approved by Alzahra University's ethical committee. Pors Online forms were used in this survey, and the link was posted on social media for participants to complete online. Prior to the distribution of the questionnaires, permission to collect data from undergraduates was obtained from the Dean of the Universities. Participants consent form was added to the first page of the questionnaire. Individuals participated in this research with satisfaction. The inclusion criteria in this study were being undergraduate students and being 18 years old or over. Participants took an average of 40 min to complete the online surveys during the data collecting period, which ran from September 2021 to December 2022.

### Measures

*The body compassion scale* (BCS; Altman et al. [14]) is a 23-item scale measuring body compassion and includes three subscales: diffusion, common humanity, and acceptance. Response options range from 1 (almost never) to 5 (almost always). The total body compassion score is calculated by summing the scores of the three subscales, with higher scores indicating higher levels of body compassion. Altman et al. [14] showed excellent internal consistency for diffusion (a = 0.92), common humanity (a = 0.91) and acceptance (a = 0.87). In the present study, Cronbach's alpha coefficients were obtained for diffusion (a = 0.91), common humanity (a = 0.93) and acceptance (a = 0.91).

*The physical appearance perfectionism scale* (PAPS; Yang and Stoeber [20]) is a 12-item measure of trait physical appearance perfectionism. The PAPS composed of two dimensions namely worry (7 items) and hope (5 items). Response options range from 1 (strongly disagree) to 5 (strongly agree) and higher scores indicate higher levels of physical appearance perfectionism. The worry about imperfection subscale was chosen for this study as it has been found to be a more maladaptive type of appearance perfectionism, whereas the hope for perfection subscale was less substantially associated with the maladaptive type of appearance perfectionism [13, 20]. McComb and Mills [6] showed excellent internal consistency for the worry about imperfection subscale (a = 0.87). In the present study, Cronbach's alpha coefficient was obtained for the worry about imperfection subscale (a = 0.89).

*The social physique anxiety* (SPA; Martin et al. [21]) is a 9-item scale used to measure the level of anxiety individuals experience when they believe others are judging their physical appearance. Response options range from 1 (not at all characteristic) to 5 (extremely characteristic), and a higher score indicates greater levels of the social physique anxiety. Woodman and Steer [22] showed acceptable internal consistency for the social physique anxiety (a = 0.9). In the present study, Cronbach's alpha coefficient was obtained for the social physique anxiety (a = 0.87).

### Statistical method

The research hypotheses in this study was answered by using a covariance-based structural equation model with AMOS software (version 24) [23]. Structural equation modeling analyzes data in three parts:

- *A: Measurement Model* Factor loading values (according to Kline [24], acceptable factor loading values are non-negative, less than 1 and greater than 0.5), measurement model fit indices (according to Byrne [25], CMIN/df < 5; Root Mean Squared Error of Approximation (RMSEA) < 0.08; Tucker–Lewis Index (TLI), Comparative Fit Index (CFI), and Goodness of Fit Index (GFI) > 0.90, indicating adequate model fit), construct reliability, and convergent validity (the values of Average Variance Extracted (AVE) and Construct Reliability (CR) were greater than 0.5 and 0.7, respectively, indicating that the measure had acceptable convergent validity and internal consistency ([25]) were assessed.
- *B: Structural Model* The beta coefficients of the linear relationships between the exogenous variables (physical appearance perfectionism and body compassion) and endogenous variable (social physique anxiety) and coefficient of determination (R^2^) were evaluated.
- *C: Moderation Analysis* Moderation analysis examines whether the moderating variable changes the strength or direction of the relationship between the exogenous variable and the endogenous variable

## Results

### Measurement model

**Fig. 1:**
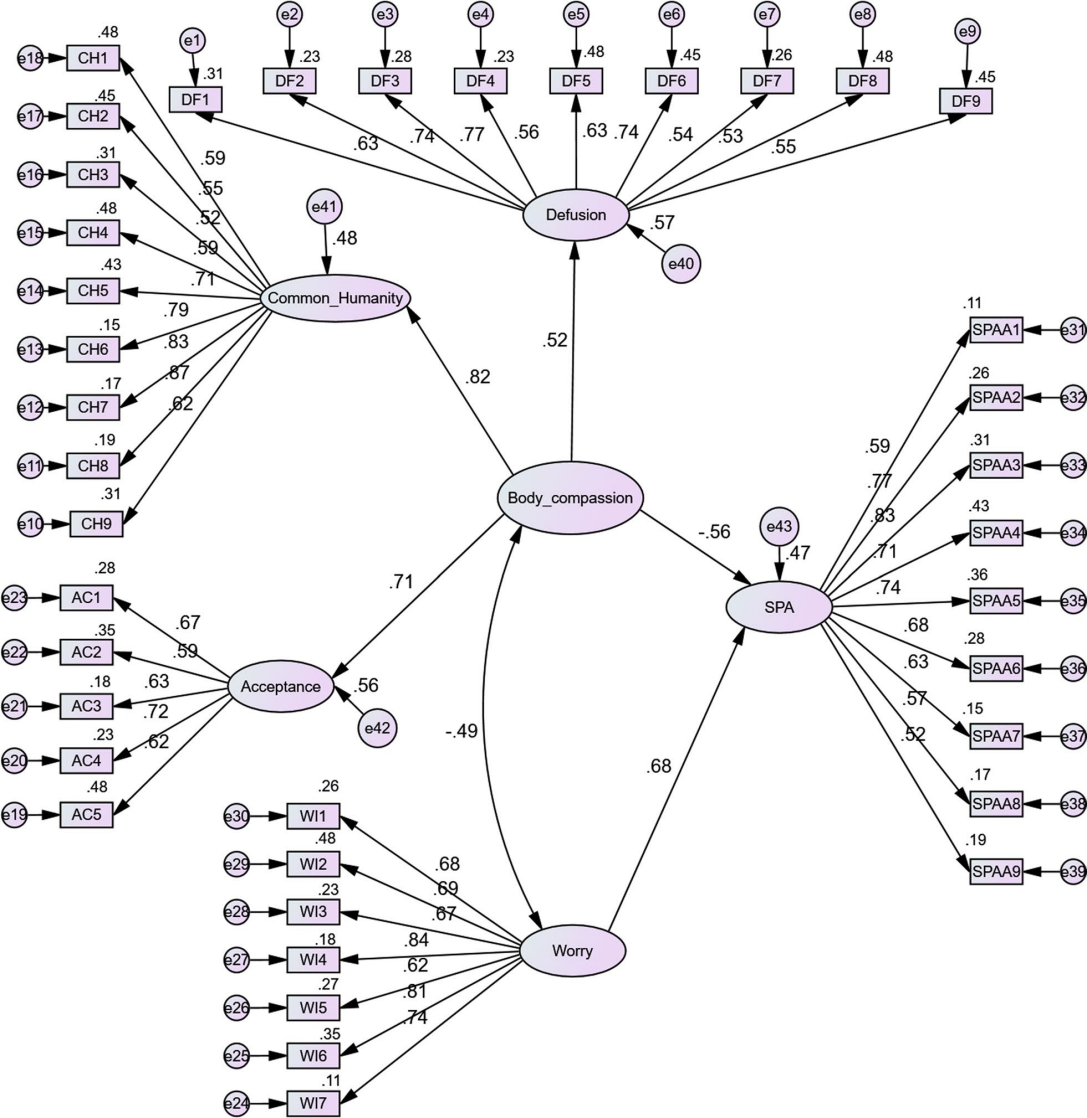
Structural model of social physique anxiety (*p* < 001)

As seen in Fig. 1, all factor loading values are less than 1 and greater than 0.5. Table 2 shows that the values of AVE and CR are larger than the cut-off scores of 0.5 and 0.7, this shows suitable convergent validity and reliability [26]. The results of the measurement model fit assessment showed that the fit indices met the cut-off values (CMIN/*df* = 3.81, *p* < 0.01, CFI = 0.94, RMSEA = 0.05, TLI = 0.94, GFI = 0.93).

**Table 2:** Values of composite reliability, average variance extracted

Variable	CR^a^	AVE^b^
Body compassion	0.82	0.71
Defusion	0.78	0.68
Common humanity	0.83	0.71
Acceptance	0.85	0.75
Worry about imperfection	0.83	0.79
Social physique anxiety	0.74	0.72

^a^Composite reliability

^b^Average variance extracted

### Structural model

The results from the structural model (see Fig. 1) showed physical appearance perfectionism (β = 0.68, *p* < 0.001) positively predicted social physique anxiety. These findings also showed body compassion (β = − 0.56, *p* < 0.001) negatively predicted social physique anxiety. To assess the variance in social physique anxiety by physical appearance perfectionism and body compassion, the coefficient of determination (R^2^) was 0.47, revealing that physical appearance perfectionism and body compassion explained 47% of the changes in social physique anxiety. This value was greater than the cut-off score value of 0.35 that is considered a moderate coefficient of determination [27].

### Moderation analysis of body compassion

A multi-group analysis was used to test the moderating role of body compassion between physical appearance perfectionism and social physique anxiety [28]. The median value (48) was used to divide body compassion as a low body compassion group with 202 (48%) participants and the high body compassion group with 216 (52%) participants.

The variant-group model and the invariant-group model should be contrasted based on fit indices in order to investigate the moderating influence of body compassion. If the fit indices for the variant-group model are better than those for the invariant-group model, this shows that the proposed model differs between the low and high body compassion groups. The body compassion variable is considered to have a moderating effect if it meets at least one of the following two criteria: (1) the path is statistically significant for one group but not for the other; or (2) the regression coefficient sign for one group is positive but not for the other group [25]. Given that the fit indices for the variant-group model (χ^2^ = 2.72, *p* < 0.01, RMSEA = 0.07, CFI = 0.91, GFI = 0.90, NFI = 0.91) were better than the fit indices of the invariant-group model (χ^2^ = 5.57, *p* < 0.01, RMSEA = 0.09, CFI = 0.75, GFI = 0.74, NFI = 0.73), it can be concluded the differences in the proposed model between the two body compassion groups. In the low body compassion group, the magnitude of relationships between physical appearance perfectionism and social physique anxiety (β = 0.79, *p* < 0.001) were greater than the high self-compassion group (β = 0.11, *p* = 0.07).

### Moderation analysis of gender

The proposed model's comparability for male and female groups was tested using a multi-group analysis. Gender groups did not moderate the proposed model, as the difference in chi square values between two models was not statistically significant ∆χ^2^ (25) = 33.77, *p* > 0.05.

## Discussion

The first goal of this study was to examine the relationships between physical appearance perfectionism, body compassion, and social physique anxiety among undergraduates. Physical appearance perfectionism was found to be a positive predictor of social physique anxiety in undergraduate students in Iran. A possible explanation for the positive relationship between physical appearance perfectionism and social physique anxiety is that individuals with high levels of physical appearance perfectionism have a negative evaluation of their body and experience higher levels of social physique anxiety because they believe that they are far from their physical ideals [29]. The results of this study are in line with earlier studies that showed individuals with high levels of physical appearance perfectionism have high beauty standards of their own body in their mind and it may not match with their physical reality and this may increase their dissatisfaction with their body [30, 31]. These individuals also have a lower sense of self-worth and suffer more from feelings of inadequacy [32]. They have extremely concern of negative evaluations by others about their physical appearance, and worry about the evaluation of others about their appearance may lead them to choice avoidant coping strategies in order not to be evaluated by others [1].

The second goal of this study was to explore whether body compassion plays a moderating role in the relationship between physical appearance perfectionism and social physique anxiety. The findings of this study showed that body compassion moderate the relationship between physical appearance perfectionism and social physique anxiety. That is, individuals with high levels of body compassion are less likely to experience social physique anxiety if they have high levels of physical appearance perfectionism.

Results of the study found that the body compassion helps individual to have a kind and non-judgmental attitude towards their bodies. Common humanity may help individuals to reduce their social physique anxiety. In other words, individuals with high levels of common humanity decrease isolation and discomfort, as it allows individuals to accept experiences and emotions without avoiding and over-identification. This also helps individuals neither avoid nor get lost in suffering of self and to have more acceptance of the suffering of self. Acceptance helps individuals to live in the present and to not be involved in the past events or future thoughts [19]. Body compassion helps individuals accept their body deficits and can help individuals to have effective social relationships, mitigate the symptoms of social physique anxiety, and may regulate emotional processes [14]. Diffusion component of body compassion helps individuals to reduce body evaluations and accept own body adequately. Diffusion also helps individuals not to identify themselves with their body's flaws, limitations, or inadequacies [19]. Based on previous studies [17, 33] combines with the results of the current study, it is confirmed that body compassion plays an important role as a significant protective factor and moderating factor between physical appearance perfectionism and social physique anxiety.

The findings of the current study demonstrated that physical appearance perfectionism may lead to social physique anxiety and body compassion acted as a moderator in this link. Therefore, psychologists could help university students in order to prevent them from social physique anxiety by assessing of physical appearance perfectionism and body compassion. Although the findings of this study are from cross-sectional study, it is conceivable that by increasing body compassion in undergraduates, physical appearance perfectionism and social physique anxiety would be decreased.

The current study included a number of limitations. First, this is a cross-sectional study and the scope and long-term validity of the findings are limited. Future studies could employ a longitudinal method to acquire more reliable data. The study sample was the study's second shortcoming. This is because the study population was made up entirely of undergraduate students from a single country, it's important not to extrapolate the findings to other groups. It is recommended that the hypothesized model in this study be studied on other age groups.

The results of this study showed that physical appearance perfectionism positively predicted social physique anxiety and body compassion negatively predicted social physique anxiety. The findings from moderation analysis showed when body compassion was added in the model, the magnitude of association between physical appearance perfectionism and social physique anxiety were reduced.

## Data Availability

The data is available on the figshare repository website https://doi.org/10.6084/m9.figshare.14068241.

## Abbreviations

SPA: Social physique anxiety
BCS: Body Compassion Scale
PAPS: Physical Appearance Perfectionism Scale
